# Pre‐ and Post‐Copulatory Sexual Traits Influence Male Fitness Across a Mosaic Hybrid Zone

**DOI:** 10.1002/ece3.70935

**Published:** 2025-02-23

**Authors:** Logan M. Maxwell, Jennifer Walsh, Brian J. Olsen, Adrienne I. Kovach

**Affiliations:** ^1^ Department of Natural Resources and the Environment University of New Hampshire Durham New Hampshire USA; ^2^ Fuller Evolutionary Biology Program Cornell Lab of Ornithology Ithaca New York USA; ^3^ School of Biology & Ecology University of Maine Orono Maine USA

**Keywords:** hybridization, mate competition, sexual selection, sperm competition

## Abstract

Primary and secondary male sexual traits can influence the interspecific interactions of hybridizing populations, yielding fitness consequences and either promoting or restricting gene flow. In this study, we evaluated the relative male fitness of two species of hybridizing tidal marsh endemics: saltmarsh ( *Ammospiza caudacutus* ) and Nelson's sparrows ( *A. nelsoni* ) and assessed the effects of male condition and competitive ability on resulting patterns of paternity and gene flow. We compared reproductive success (number of offspring sired) among saltmarsh, Nelson's, and hybrid sparrow males (*n* = 125) and modeled male fitness in relation to measured pre‐copulatory (body size, fat scores, and muscle scores) and post‐copulatory (cloacal protuberance (CP) volume and sperm length) male sexual traits across two sites within the center of the hybrid zone. We found saltmarsh sparrows had higher levels of skew in fertilization success than Nelson's and greater reproductive output than both Nelson's and hybrids, suggesting interspecific competition may occur. Body size was the best predictor of reproductive success, independent of male genotypes, providing evidence for a role of pre‐copulatory sexual selection. We also found evidence of post‐copulatory sexual selection and sperm competition contributing to patterns of hybridization, with CP volume and sperm length increasing with number of offspring sired. Differential mean fitness by species may influence patterns of hybridization and has the potential to drive asymmetrical introgression; however, the drivers of male fitness differed between species and sites, suggesting the level of sexual selection and resulting patterns of gene flow are context dependent and not stable across a small sptatial scale within the center of this mosaic hybrid zone. Overall, few interspecific offspring and nearly equal backcrossing in both parental species within the center of the hybrid zone suggest mechanisms such as reinforcement exist to limit hybridization and minimize asymmetric introgression.

## Graphical abstract

We used SNP genotypes to assess and compare male reproductive success in two inter‐breeding species of marsh sparrow—saltmarsh and Nelson's sparrow—and their hybrids. Saltmarsh sparrow males had higher reproductive output, greater variance in reproductive success, and higher levels of multiple paternity than Nelson's sparrows and hybrids. We found evidence for both pre‐copulatory (body size) and post‐copulatory (sperm competition) sexual selection, with drivers of fitness differing between the two species. Photo shows a pair of mating saltmarsh sparrows (credit: Grace McCulloch).

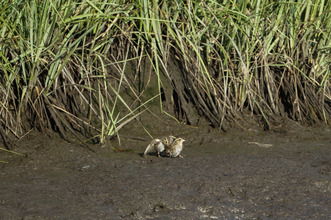

## 1 Introduction

Sexual traits are known to influence mating behaviors and interspecific interactions of hybridizing populations, either promoting gene flow between species or impeding it through reproductive isolation (Irwin and Price 1999). This role of sexual selection on hybridization and speciation is often framed in the context of female mate choice and male–male competition, whereby females, due to higher investment in gametes, choose mates based on inferred reproductive benefits or consequences of hybridization. Males, therefore, may not be as choosy if they mate intra or interspecifically, as they maximize fitness by mating frequently with multiple females (Grant and Grant 1997; Parker and Partridge 1998).

When hybridization is maladaptive, reinforcement may evolve—whereby divergence in mating preference and sexually selected traits may increase avoidance of between‐species mating (Schumer et al. 2017; Servedio and Noor 2003). Although this explains mechanisms by which female mate choice can drive divergence, Darwin's second mechanism of sexual selection, male–male competition, can also play a role (Darwin 1871). Male–male competition is a form of intrasexual selection, in which interactions among males drive sexual selection due to competitive access to females (Darwin 1871; Andersson 1994). In many systems, it can generate strong selection that favors divergent phenotypes between species and competition for mating resources, which can in turn drive speciation via natural selection (Lackey et al. 2018). Ultimately, interactions between female choice and male competition are likely important in divergence and speciation in the face of gene flow (Van Doorn et al. 2004; Lackey et al. 2018; Lipshutz 2018; Wong and Candolin 2005).

Intersexual mate choice and intrasexual competition for mates can lead to variance in fitness, which drives selection (Fisher et al. 2016) and is often responsible for the evolution of numerous kinds of male secondary sexual traits or sexual signals involved in pre‐copulatory competition (Andersson 1994). These traits can be static, like some body morphometrics, but can also be more plastic, such as behavior. Avian sexual characteristics involved in competition include ones directly used in fighting, including body size, as well as traits important in dominance and signaling, such as song or mate guarding (Andersson 1994; Hagelin 2002).

Post‐copulatory sexual selection may also be a powerful selective agent, but generally involves primary, as opposed to secondary, sexual characteristics. For instance, sperm competition, an intense form of post‐copulatory male–male competition, occurs when sperm from males compete for the fertilization of eggs within a female's reproductive tract (Parker 1970) and increases the relative variance in male mating success (Moller and Ninni 1998). Higher sperm production may lead to higher male fitness, because this can allow males to copulate more often or release more sperm per ejaculate, while increased sperm size or motility might enhance an individual male's ability to fertilize eggs (Laskemoen et al. 2010). However, direct effects of sperm morphology may be masked by cryptic female choice, whereby females are able to influence fertilization success (Birkhead 1998). Primary sexual characters can often be hard to study in natural populations, without invasive sampling or controlled experiments; however, in birds there are some morphometric cues that can help determine the role of sperm competition in mating success. The intensity of sperm competition is one factor known to determine variation in the size of male reproductive organs in birds (Sax and Hoi 1998). Accordingly, the size (volume) of the cloacal protuberance (CP) has been found to be a proxy for sperm production, sperm velocity, and fertilization success (Laskemoen et al. 2008, 2010; Peer et al. 2000; Tuttle et al. 1996); accordingly, CP size may serve as a proxy for sperm competition.

Mate competition in secondary contact may promote reproductive isolation in the form of reproductive or agonistic character displacement, where divergence in competitive traits or mating signals reduce interspecific interactions (Lipshutz 2018). However, interspecific interactions in secondary contact are not limited to reducing gene flow through reproductive isolation, rather these interactions can also promote hybridization in some circumstances (Lipshutz 2018; Veen et al. 2001). Interspecific reproductive competition may occur when species compete for limited space in relation to mate attraction and reproduction (Grether et al. 2009; Martin et al. 2017). This can lead to increased introgression if males of one species are more dominant on average than the other (Krosby and Rohwer 2010; Pearson 2000). Competitive asymmetry can promote directional hybridization, and in some cases generate asymmetric introgression from the competitively superior species to the inferior (While et al. 2015). These dynamics are not always stable within hybrid zones, as processes that influence the exchange of genetic variation can differ on fine spatial scales depending on spatial groupings of individuals or the mosaic of suitable microhabitat (Zonana et al. 2021; Shurtliff et al. 2014).

In this study, we evaluated relative male fitness (number of offspring sired) in two hybridizing species of tidal marsh endemic sparrows: saltmarsh (Ammospiza caudacutus) and Nelson's sparrows (A. nelsoni). We further assessed the relative influence of pre‐ and post‐copulatory sexual traits on patterns of paternity and resulting gene flow. The sister species are currently in secondary contact, and form a mosaic hybrid zone where their populations co‐occur on tidal salt marshes in the northeastern coast of the United States from South Thomaston, Maine, USA to Plum Island in Newburyport, Massachusetts, USA (Hodgman et al. 2002; Shriver et al. 2005; Walsh, Rowe, et al. 2015). Previous work in the southern portion of the saltmarsh—Nelson's sparrow hybrid zone documented high promiscuity and reproductive skew in both saltmarsh and Nelson's sparrow males, with evidence for decreased fitness in hybrid males (Walsh et al. 2018). However, these mating behaviors may not be reflective of reproductive strategies in sympatric populations near the center of the hybrid zone where the densities of the two species are more equal. Indeed, previous studies have shown that population sizes, genotypic composition, and selective pressures vary on a fine spatial scale across sites within the center of the hybrid zone, with coastal sites being larger, having more saltmarsh sparrows and introgression towards them, and stronger signals of sexual selection than their inland counterparts (Maxwell et al. 2021). The role of male sexual characteristics in driving patterns of mating, fitness, and consequently, hybridization between these two species, however, is still unknown.

Closely related species often show more divergence in secondary sexual characteristics than other phenotypic traits (Allender et al. 2003). Morphologically, Nelson sparrows tend to be smaller than saltmarsh sparrows (e.g., bill length, weight), and have paler and less discrete plumage characteristics (Greenlaw 1993; Shriver et al. 2005). Both sparrows exhibit a polygynandrous mating system, which is nearly unique among songbirds, in which males are non‐territorial, provide no parental care to young (Greenlaw 1993; Shriver et al. 2007), and exhibit high levels of multiple paternity (Hill et al. 2010; Walsh et al. 2018). Saltmarsh sparrow males, however, engage in scramble‐competition to gain access to females and spend significant time searching for and pursing females as well as chasing off and fighting with other males. Conversely, Nelson's sparrows may guard females during their fertile period and spend more time producing sexual displays, including songs from both perches and during sometimes‐frequent flight displays, the latter of which are rare or absent in saltmarsh sparrows (Greenlaw 1993; Shriver et al. 2007, 2010). Due to the nature of the scramble polygynandrous mating system and greater sperm length in saltmarsh relative to Nelson's sparrows (Cramer et al. 2021), it is predicted that post‐copulatory processes may also drive patterns of fertilization. Thus there are reasons to believe that differences in both pre‐ and post‐copulatory processes between the species could influence patterns of gene flow and hybridization in saltmarsh and Nelson's sparrows.

### 1.1 Objectives & Predictions

In this study we aimed to determine:

1. The relative fitness and reproductive skew among saltmarsh sparrow, Nelson's sparrow, and hybrid males across two sympatric sites within the center of the hybrid zone.
  We expect short‐term relative fitness (number of offspring sired) and reproductive skew (unequal partitioning of reproduction among males) to be a function of genotype such that either:
  - Saltmarsh sparrows will have greater fitness and more reproductive skew than Nelson's sparrows, with hybrids intermediate, due to differences in size, behavior, and mating strategy. Accordingly, males with higher proportion of saltmarsh sparrow alleles will be more successful because they will be competitively superior in a scramble competition for mates and fare better using this strategy than by mate‐guarding; they may also have an advantage through direct or indirect female choice.
  - Or, both saltmarsh and Nelson's sparrows will have greater fitness and reproductive skew than hybrids who will not perform well in either mating strategy nor be a successful competitor for mates due to intermediacy in species‐specific mating behaviors and morphological characteristics.
2. The influence of pre‐ and post‐copulatory sexual traits on observed male fitness.
  We expect pre‐copulatory condition (mass, fat, and muscle scores) and post‐copulatory sexual characteristics (CP volume and sperm length) to influence differential fitness (number of offspring sired) among all males, regardless of genotype. Specifically, we predict that pre‐ and post‐copulatory sexual characteristics of condition and competitive ability of all males will be positively correlated with reproductive success due to sexual selection.
3. Differences in drivers of male fitness across sites within the center of the hybrid zone.
  We also expect the relative influence of pre‐ and post‐copulatory drivers of male relative fitness will differ across two study sites within the center of the hybrid zone. Given variation in genotypic composition, strength of sexual selection, and patterns of introgression at small spatial scales within this hybrid zone (with a coastal site having more saltmarsh sparrows and showing stronger sexual selection than an inland site), we expect different combinations of pre‐ and post‐copulatory male sexual traits to predict number of offspring sired at the two different sites.

## 2 Methods

Data used in this study were collected as part of previously published work assessing patterns of introgression across the saltmarsh‐Nelson's sparrow hybrid zone, documented in Maxwell et al. (2021). Here, we combine previously reported paternity and mate reconstruction data with male reproductive success and condition metrics to assess male fitness and sexual selection.

### 2.1 Study Area & Field Data Collection

**FIGURE 1:**
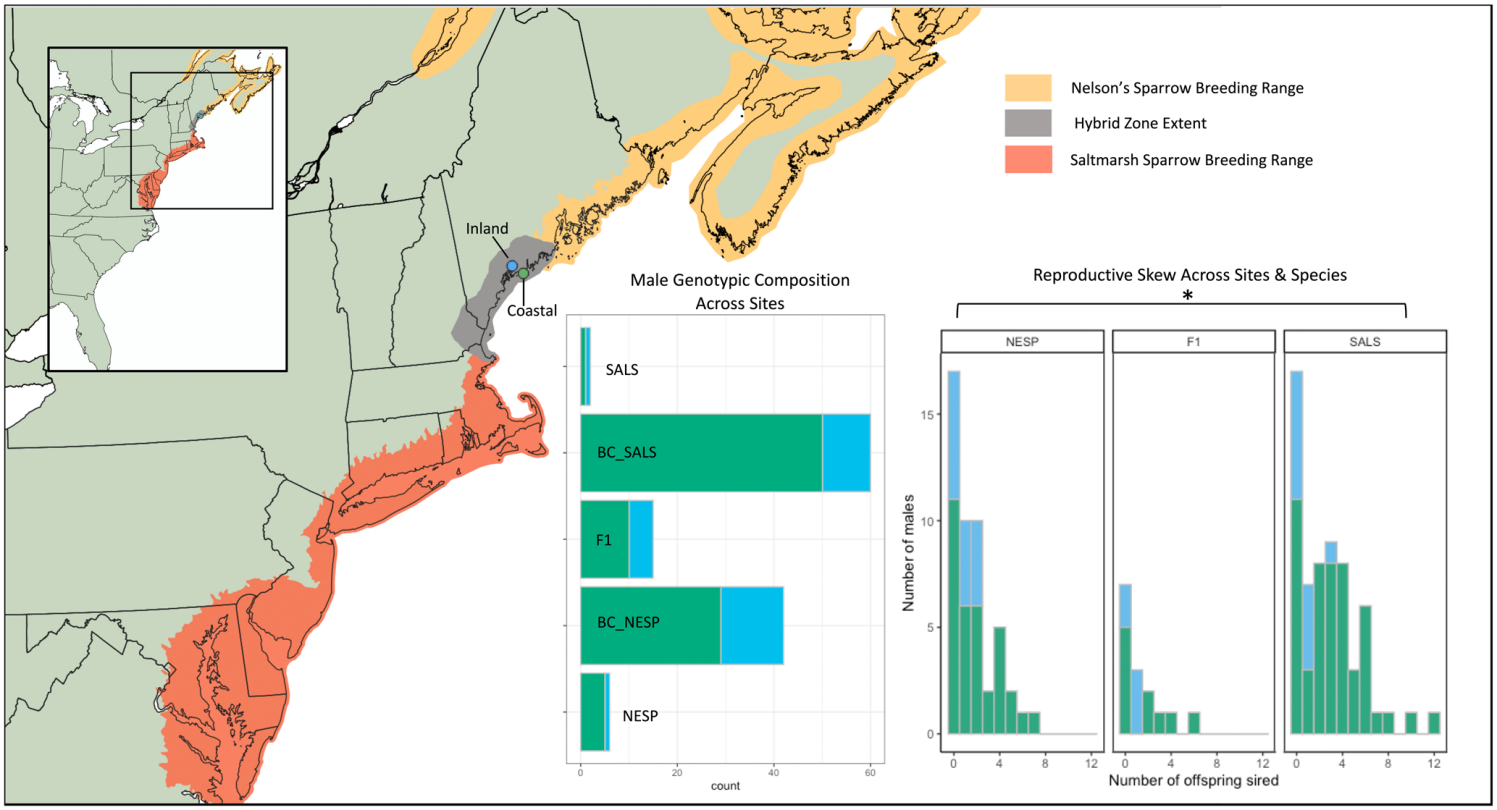
Sampling location map showing both the inland (blue) and coastal (green) sites at which male Nelson's, saltmarsh, and hybrid sparrows were sampled and monitored across 2016 and 2017 breeding seasons. Reproductive skew (distribution of offspring sired per male) for each species and at each site (inland = blue, coastal = green) are displayed in the paneled histogram, with asterisk denoting significance at the 0.05 alpha level. The genotypic makeup of male captured at each site are displayed in a second paneled histogram (inland = blue, coastal = green). Species breeding range extent are shaded in orange for saltmarsh sparrow and yellow for Nelson's sparrow, with the hybrid zone highlighted in gray.

Sparrows were monitored at two field sites along the Northeastern U.S. coast across the 2016 and 2017 breeding seasons. These sites, located in Phippsburg and Brunswick Maine, USA are both in the center of the hybrid zone but differ in size, habitat, and amount of tidal inundation (Figure 1). Spanning opposing ends of an inland‐coastal habitat gradient, the marshes at Popham Beach State Park in Phippsburg (referred to throughout as the ‘coastal’ site) are a larger marsh complex (~15‐ha study plot) located directly on the coast, while the marsh at Wharton Point on Maquoit Bay in Brunswick (referred to throughout as the ‘inland’ site) is a third of the size of the coastal site (~5‐ha study plot) and located along a cove farther inland surrounded by forest and fields (7 km inland from the mouth of Maquoit Bay and 20 km inland from the Gulf of Maine). Daily and monthly tidal inundation is dampened at the inland site compared to that of the coastal site, which has been shown to be a major driver of female fitness and nest success (Maxwell et al. 2023), with observed differences in selective pressures and genotypic makeup between the sites (Maxwell et al. 2021). The inland site has more Nelson's sparrows and higher proportion of hybrids than the coastal site which is more saltmarsh sparrow dominated (52% of adults at inland site have Nelson's sparrow ancestry, 31% saltmarsh sparrow ancestry, and 17% hybrid, while 37% of adults at coastal site are Nelson's, 53% are saltmarsh, and 10% are hybrids; Maxwell et al. 2021). The two sites, and their differences in size and genetic makeup, are indicative of regional patterns within this mosaic hybrid zone, providing a functional sample at the population‐level for the center of the hybrid zone. Attention was given to an equal sampling effort to ensure that data collected were complete and indicative of the local conditions.

To collect male condition data, we sampled the population of sparrows at both sites with opportunistic mist netting (using 2–6, 12‐m mist nets) throughout the 2016 and 2017 breeding seasons to capture as many adult males as possible. Since the polygyandrous mating system of these sparrow species means there are no territories or social pair bonds, effort was made to ensure we sampled the entirety of the marsh area where these species co‐exist, compete for mates, and nest. Males were banded with a USGS aluminum band, a site‐specific color band, and a blood sample obtained from the cutaneous ulnar vein (~10–20 μL) and stored on Nobuto Filter Paper (Sterlitech, Kent, Washington) for determining each male's genotype and for genetic parentage analysis. We collected measurements to assess competitive ability of males, including CP size, mass, fat scores, and muscle scores. The size of the cloacal protuberance was measured by depth distal to proximal along the axial plane and width at the widest point along the sagittal plane superior to inferior at the widest section of the CP. CP volume was then calculated following Schut et al. (2012) adapted from Mulder & Cockburn (1993), via volume of a barrel (π × radius^2^ × height), with the radius calculated as 0.5 of the width at the widest point. Abdominal and furcular fat scores (0–6), as well as pectoral muscle scores (0–6) was estimated at each capture following the protocol from Danner (2012), as in Borowske et al. (2018). Individual male fat scores were calculated as an average between abdominal and furcular scores. If an individual male was captured more than once, muscle scores, fat scores and CP measurements were averaged to obtain a single measurement for each male. Sperm ejaculates via cloacal massage were opportunistically collected on males caught via mist net throughout the two breeding seasons and were mixed with 10–20 μL PBS and transferred to 10% formalin for storage (Cramer et al. 2021).

To determine the number of offspring sired from each male, we conducted nest monitoring across both years (described in Maxwell et al. 2021, 2023) and obtained blood samples from as many offspring as possible to reconstruct parentage. When nestlings were 6 days of age, they were banded with a USGS aluminum leg band and a single site‐specific color band, and a few drops of blood were taken from the medial metatarsal vein and transferred to Nobuto Filter Paper for genetic parentage analysis. We also collected many dead, unbanded chicks following a nest flooding event or any eggs that failed to hatch and included these in our paternity analyses (flooding is the primary cause of nest failure in this system and flooded nests with drowned chicks are common). To determine the identity of females associated with each nest (as needed for subsequent parentage analyses), we conducted targeted mist‐netting to capture females off their nests during incubation or brooding, as described in Maxwell et al. (2023). Once caught, each female was banded with a USGS aluminum band, a site‐specific color band, and a Passive Integrated Transponder (PIT; Biomark, Boise, ID) tag that was modified to a color band for non‐invasive detection of re‐nesting attempts. A blood sample was drawn from the cutaneous ulnar vein (~10–20 μL) and stored on Nobuto Filter Paper for use in paternity analyses and determining the genotype of the nesting female.

### 2.2 Assigning Genotypic Classes

The genotypes of all sampled adults from the two field seasons were previously determined using 135 single nucleotide polymorphisms (SNPs) identified as fixed between the species from double digest restriction site associated DNA (ddRAD) sequencing libraries, as described in Maxwell et al. (2021, 2023). Briefly, the 135 fixed SNPs (identified from allopatric samples of each species) were used to determine the genetic ancestry of individuals using methods of Milne and Abbott (2008) that combines hybrid index and interspecific heterozygosity to place individuals into genotypic classes. Hybrid index was defined as the proportion of alleles inherited from the saltmarsh sparrow (0 = pure Nelson's sparrow and 1 = pure saltmarsh sparrow), and interspecific heterozygosity is the proportion of genotypes that are heterozygous across the species for the parental alleles (0 = all homozygous genotypes, found only in one parental species, and 1 = all heterozygous genotypes across species). We placed all individuals into one of five genotypic classes: pure Nelson's sparrow, backcrossed Nelson's, F1/F2, backcrossed saltmarsh, or pure saltmarsh sparrow. Individuals with intermediate hybrid index (0.25–0.75) and high heterozygosity (> 0.3) were considered recent generation hybrids (F1 or F2), and individuals with very low or high hybrid index (0.05–0.25 or 0.75–0.95) and low heterozygosity (< 0.3) were considered backcrossed. Pure individuals are defined as a hybrid index of 0–0.05 (Nelson's sparrow) or 0.95–1 (saltmarsh sparrow). Due to the small sample sizes of pure individuals, we categorized all individuals into three broad genotypic classes for subsequent analyses: backcrossed and pure Nelson's sparrows, backcrossed and pure saltmarsh sparrows, and recent generation hybrids (F1, F2). All statistical analyzes were performed in program R with packages and functions named in each section below where the analysis is described (R Core Team 2022).

### 2.3 Paternity Analyses

We determined the number of offspring sired by each male through paternity analyses of nestlings with known mothers (Maxwell et al. 2021). A stringently filtered set of 589 SNPs identified from ddRAD libraries was used to assign candidate fathers to each nestling using two methods: CERVUS (Marshall et al. 1998) and COLONY v2.0 (Jones and Wang 2010). Because the likelihood approach of CERVUS does not account for unsampled males in the population, COLONY was also used, because it can determine the number of sires per nest, even if the father was not sampled. Input parameters included genotyping error rate of 1%, 95% of loci typed, candidate father sampling of 70%, and we assumed the proportion of sampled mothers to be 95%. Analyses were done separately for each site (inland and coastal) and each breeding season (2016 & 2017). For the second year of the study (2017) we included all sampled males from both breeding seasons in the paternity analyses (adults and offspring as determined from molecular sexing) to account for any returning adult males that may have evaded capture in the second season of the study. We compared the most likely father as assigned by CERVUS (delta trio value ≥ 95%) to that made in COLONY for each nestling individually. For any discrepancies on confident paternity assignments (> 95%) we manually assessed the number of loci mismatches, delta pair confidence, and overall loci typed to identify the best assignment.

### 2.4 Relative Fitness and Reproductive Skew Across Genotypes

To determine if male reproductive success was a function of genotype (i.e., the two species groups and recent hybrids), we evaluated number of offspring sired in relation to genotypic class of each male across the two breeding seasons. We modeled number of offspring sired across the three genotypic classes (saltmarsh, Nelson's and hybrids) using poisson regression, (due to count nature of the data) and included site as a covariate, since genotypic frequencies differed between sites (‘*glm*’ function in stats package in R). We followed the modeling with a post hoc pairwise comparison tests with the Tukey method (‘*pairs*’ function in emmeans package) to identify differences in male reproductive success (number of offspring sired) among the three groups of males (saltmarsh, Nelson's, hybrids). To determine if fitness trends across genotypes met our specific predictions, we modeled the number of offspring sired in relation to hybrid index values for each male (0–1; proportion of alleles inherited from saltmarsh sparrow) using both a linear Poisson regression (given non‐normal nature of the count data) and quadratic distribution, both including site as a covariate. Support for a linear model would suggest hybrids were intermediate in relation to parental genotypes (prediction 1a), while support for the quadratic model would suggest hybrids had lower reproductive success than both parental genotypes (prediction 1b).

To assess differences in reproductive skew between the species, we determined the number of offspring sired per individual male and compared this distribution across all genotypes. We used a two‐sample Komogorov–Smirnov test (function ‘*ks. test*’ program stats) to determine if there were significant differences in number of offspring sired per male among saltmarsh, Nelson's and hybrid sparrows. Differences in reproductive skew were also evaluated between the two sampling sites; however, small sample sizes at the inland site precluded our ability to test for differences statistically.

We looked for differences in levels of multiple paternity among the three genotypic classes of Nelson's, saltmarsh, and hybrid sparrows. To do so, we modeled the number of nests at which an individual male sired offspring across the genotypic classes using poisson regression (due to the count‐nature of the data) across sites, and with site as a covariate. In this way, we could assess the average impact of species across sites within the region in addition to determining site differences. Post hoc comparative tests were performed with the Tukey method for establishing differences among the genotypic classes. We also tested for an association between the number of nests at which a male sired offspring and its hybrid index using Poisson regression.

### 2.5 Pre‐ and Post‐Copulatory Drivers of Fitness

To address our second objective and determine if male pre‐copulatory condition and post‐copulatory competitive ability were positively correlated with reproductive success (regardless of genotype) at the population‐level, we first modeled number of offspring sired in relation to four measured male sexual traits: CP volume, mass, fat scores, and muscle scores. We subsetted our data to only those males for which we had complete data across the condition metrics across the 2 years and used the DHARMa package in program R to check distribution assumptions and residuals (total of 96 individual males). Since the data were found to be over‐dispersed (variance exceeded the mean) and did not meet the assumptions of Poisson regression (commonly used to model count data), we opted to perform negative binomial regression. Negative binomial regression allows for greater flexibility in model fitting, with an additional parameter (k) that accounts for clumping and has been found to be useful with count data where there is an excess of zeros in the dataset. We first tested for correlations between our four predictor variables using the ‘*cor.test*’ function in package stats in program R. We found a significant correlation between mass and CP volume (Pearson's correlation = 0.47; *p* = 1.^2−^6), as well as muscle scores and fat scores (Pearson's correlation = 0.20; *p* = 0.05); however, given the relatively low correlation coefficients, we retained all variables in our modeling. There were no significant correlations between any of the other four predictor variables. We then used the ‘*dredge*’ function in the MuMIn package in program R for automated model selection within an AIC framework. We compared all additive models of reproductive success using function ‘*glm.nb*’ in package MASS across the four measured male predictor variables. For any condition metric we found to be an informative predictor of male reproductive success across all genotypes (confidence intervals on the beta estimates did not overlap zero), we subsequently performed univariate tests for this pattern within species, using negative binomial regression. In this way, we could assess if sexual selection was operating the same within as across species.

To evaluate post‐copulatory drivers of fitness, we used previously analyzed morphometrics of sperm samples from a subset of 27 males in this study for which we also have reproductive success and CP volume data. The sperm morphology metric of overall sperm length (μm) from Cramer et al. (2021) was used. These data were used in our analyses in two ways: (1) to assess the validity of using CP volume as a proxy for sperm competition, and (2) to determine if there was a relationship between overall sperm length and the number of offspring sired. We used linear regression to test for the relationship between each male's observed CP volume and average total sperm length using the ‘*glm*’ function in the stats package in program R and visualized this relationship across the males genotype (saltmarsh, Nelson's, or hybrid) using the ggplot2 package in program R. A significantly positive correlation between sperm length and CP volume (regardless of genotype) would provide further support for the use of CP volume as a proxy for sperm competition in this system. Subsequently, we used Poisson regression (‘*glm*’ function in R package stats) to test for a relationship between the number of offspring sired and the average total sperm length for each male. To further assess whether observed patterns were due to species differences, we visualized this relationship using ggplot2 package in R across saltmarsh, Nelson's and hybrid males. Due to small sample sizes, we were unable to formally test for differences between or within species/hybrids.

### 2.6 Site‐ Specific Drivers of Fitness

To address our third objective and because previous work has shown differences in relative influence of sexual selection between our two study sites (Maxwell et al. 2021), we additionally modeled male reproductive success at each site separately with the same four predictor variables (mass, CP volume, fat scores, and muscle scores). We followed the same methods as above (across sites), using negative binomial regression (package MASS; function ‘*glm.nb*’) in an AIC framework and compared all additive models across the predictor variables using the ‘*dredge*’ function in the MuMIn package in R.

To further understand the differences across the two sites, we looked at the observed genotypic classes (male and female) of successful mate pairs from paternity assignments to determine if any patterns exist broadly in the center of the hybrid zone and if they are consistent between sites. Each successful mating event was categorized into 1 of 9 possible combinations: (1) hybrid female with hybrid male; (2) hybrid female with Nelson's male; (3) Nelson's female with hybrid male; (4) Nelson's female with Nelson's male; (5) Nelson's female with saltmarsh male; (6) saltmarsh female with Nelson's male; (7) saltmarsh female with saltmarsh male; (8) hybrid female with saltmarsh male; and (9) saltmarsh female with hybrid male. Previously we found patterns of assortative mating at the coastal but not inland site (Maxwell et al. 2021), but here our aim was to assess if there was one specific genotypic mate pairing that dominates successful mating attempts and drives patterns of sexual selection, by comparing the proportion of successful mating attempts across the nine potential mate pair categories.

To account for mate availability, an expected random distribution of pairings across all categories was determined through a contingency table based upon observed frequencies of adult male and female genotypic classes both across and within sites. In this way, we could compare observed mating pairs with those predicted by random mating across the available genotypes. We tested for significance between observed and theoretical distribution of mate pairs using a Fisher Exact test (function ‘*fisher.test*’), and further tested for which mate pair categories were significantly different from theoretical distributions using a Fisher Exact test with holm *p*‐value correction for multiple comparisons (function ‘*row_wise_fisher_test*’ in package rstatix) in program R.

## 3 Results

Across our two study sites, we genotyped a total of 125 adult male birds. Using the hybrid index and interspecific heterozygosity, we determined that 48% were backcrossed saltmarsh sparrows (60 individuals), 33% of the adult males were backcrossed Nelson's Sparrows (42 individuals), 12% were recent generation hybrids (F1/F2; 15 individuals), 5% were pure Nelson's sparrows (6 individuals), and 2% were pure saltmarsh sparrows (2 individuals; Figure 1). Due to low sample sizes, we binned backcrossed and pure individuals for subsequent analyses, which resulted in 50% saltmarsh and backcrossed saltmarsh sparrow individuals (62), 38% Nelson's and backcrossed Nelson's individuals (48), and 12% recent‐generation hybrids individuals (15). Of the 125 adult male birds, 24% (30 individuals) resided at the inland site (10 backcrossed saltmarsh sparrows, 1 pure saltmarsh sparrow, 13 backcrossed Nelson's sparrow, 1 pure Nelson's sparrow, and 5 F1/F2) while 76% (95 individuals) resided at the coastal site (50 backcrossed saltmarsh sparrows, 1 pure saltmarsh sparrow, 29 backcrossed Nelson's sparrows, 5 pure Nelson's sparrows, and 10 F1/F2; Figure 1).

We monitored a total of 201 nests across the 2016 and 2017 breeding seasons and were able to genotype 301 sampled nestlings/collected eggs. We assigned paternity to 274 of the 301 offspring (91%) genotyped. Paternity assignments were in 100% agreement between COLONY and CERVUS at the inland Site. At the coastal site there were 13 cases in which COLONY and CERVUS did not agree. For all 13 of these instances, CERVUS had either high loci mismatches or low delta pair confidence levels, and COLONY provided higher confidence; therefore, we used the COLONY assignments for these cases.

### 3.1 Relative Fitness and Reproductive Skew Across Genotypes

Overall, we found site (*z* = −2.07; *p* = 1.2 ^−7^) and genotypic class (*z* = 0.59; *p* = 0.01) were significant predictors of the number of offspring sired (Poisson Regression). Post hoc Tukey tests showed pure and backcrossed Nelson's sparrows sired fewer offspring than pure and backcrossed saltmarsh sparrows (estimate = −0.41; *p* = 0.007), while hybrids sired an equal number of offspring to Nelson's sparrows (estimate = −0.17; *p* = 0.77) and fewer offspring than saltmarsh sparrows (estimate = −0.59; *p* = 0.034). There was more support for the linear model of number of offspring sired with hybrid index and site than that of the quadratic (linear term estimate 4.96; *p* = 0.02 vs. quadratic term estimate 2.48; *p* = 0.23). Linear Poisson regression showed both site (*z* = 1.20; *p* = 6.9^−8^) and hybrid index (*z* = 0.55; *p* = 0.001) were significant predictors of number of offspring sired.

There were many sampled males that produced no offspring for the duration of the study (33%; Table 1). By broad genotypic class, approximately half (46%) of the hybrid adult males, 36% of Nelson's sparrows (pure and backcrossed), and 27% of saltmarsh sparrow (pure and backcrossed) males sired no offspring (Table 1). Of all the males that sired at least one offspring (67%), 54% were backcrossed saltmarsh sparrow, 37% were backcrossed Nelson's sparrow, 9% were hybrids. Generally, patterns of paternity showed that saltmarsh sparrow males sired more offspring and had more variation in the number of offspring sired across males than Nelson's and hybrids (Figure 1; Table 1). Of all the birds that sired at least one offspring, the majority of hybrids and Nelson's sparrows sired 1 or 2 offspring, while the majority of saltmarsh sparrows sired 3 or more offspring (Table 1). Thirteen male saltmarsh sparrows (21%) produced 5 or more offspring (with a maximum of 12), while most males (66%) produced only 0–3 offspring over the 2 years (27% produced 0 offspring and 39% produced 1–3 offspring; Table 1). We tested for differences in reproductive skew among the species and hybrids (distribution of offspring sired per male; Figure 1) and found that saltmarsh sparrows had higher levels of reproductive skew than Nelson's sparrows (*D* = 0.255; *p* = 0.02), while there was no difference between saltmarsh sparrows and hybrids (*D* = 0.234; *p* = 0.14) or Nelson's sparrow and hybrids (*D* = 0.113; *p* = 0.086). Patterns of reproductive skew were consistent across the study sites; however, skew was less pronounced at the inland site with slightly more equal levels of reproductive success regardless of genotype (Figure 1), although we did not formally test this relationship due to small sample sizes at the inland site.

**TABLE 1:** Observed reproductive skew of Nelson's, saltmarsh, and hybrid sparrows across both sites and sampling years, showing the percentage (number) of males that produced 0,1,2,3, 4, and > 5 offspring.

	Sired 0 offspring	Sired 1 offspring	Sired 2 offspring	Sired 3 offspring	Sired 4 offspring	Sired 5+ offspring
Hybrids (F1/F2)	46% (7)	20% (3)	13% (2)	7% (1)	7% (1)	7% (1)
SALS (backcrossed and pure)	27% (17)	11% (7)	13% (8)	15% (9)	13% (8)	21% (13)
NESP (backcrossed and pure)	36% (17)	21% (10)	21% (10)	4% (2)	10% (5)	8% (4)

We observed high levels of multiple paternity across all genotypic classes. After excluding nests that had only 1 chick (leaving 80 nests in total), we found that 28 nests (35%) had a different father for each chick, while only 15 (19%) of nests had only one father. Over half of all nests (54%) had two fathers, and 15 (19%) had three fathers. Of the 28 nests with a different father for each chick, 15 (54%) were saltmarsh sparrow female nests, 8 (29%) were Nelson's sparrow female nests, and 5 (18%) were hybrid female nests. Additionally, we found genotype to be a significant predictor of the number of nests from which males sired offspring in the absence of site as a co‐variate (*z* = 1.99; *p* = 0.05), with post hoc tests illustrating that at the population level, Nelson's sparrows sired offspring from fewer nests than saltmarsh sparrows (estimate = −0.50; *p* = 0.005), and no differences between hybrids and either parental species (estimate = −0.50, *p* = 0.11 for F1 and saltmarsh; estimate = −0.007, *p* = 0.99 for F1 and Nelson's). The model in which we included site as a covariate with genotype resulted in site as a significant predictor of number of nests from which offspring were sired (*z* = 4.09; *p* = 4.43^−5^), but not genotype (*z* = 1.53; *p* = 0.13). We also found a significant positive relationship between male hybrid index values and the number of nests from which they sired offspring (Poisson regression; β = 0.66 ± 0.19; *z* = 3.39; *p* = 0.0007), indicating that birds with more saltmarsh sparrow alleles not only sire more offspring, but also do so from a greater number of nests. This positive relationship between hybrid index and the number nests from which offspring were sired held true when we included site as a covariate in the model with hybrid index (*z* = 2.9; *p* = 0.004), while site (*z* = 4.13; *p* = 4.68^−5^) was also found to be a significant predictor.

### 3.2 Pre‐ and Post‐Copulatory Drivers of Fitness

**FIGURE 2:**
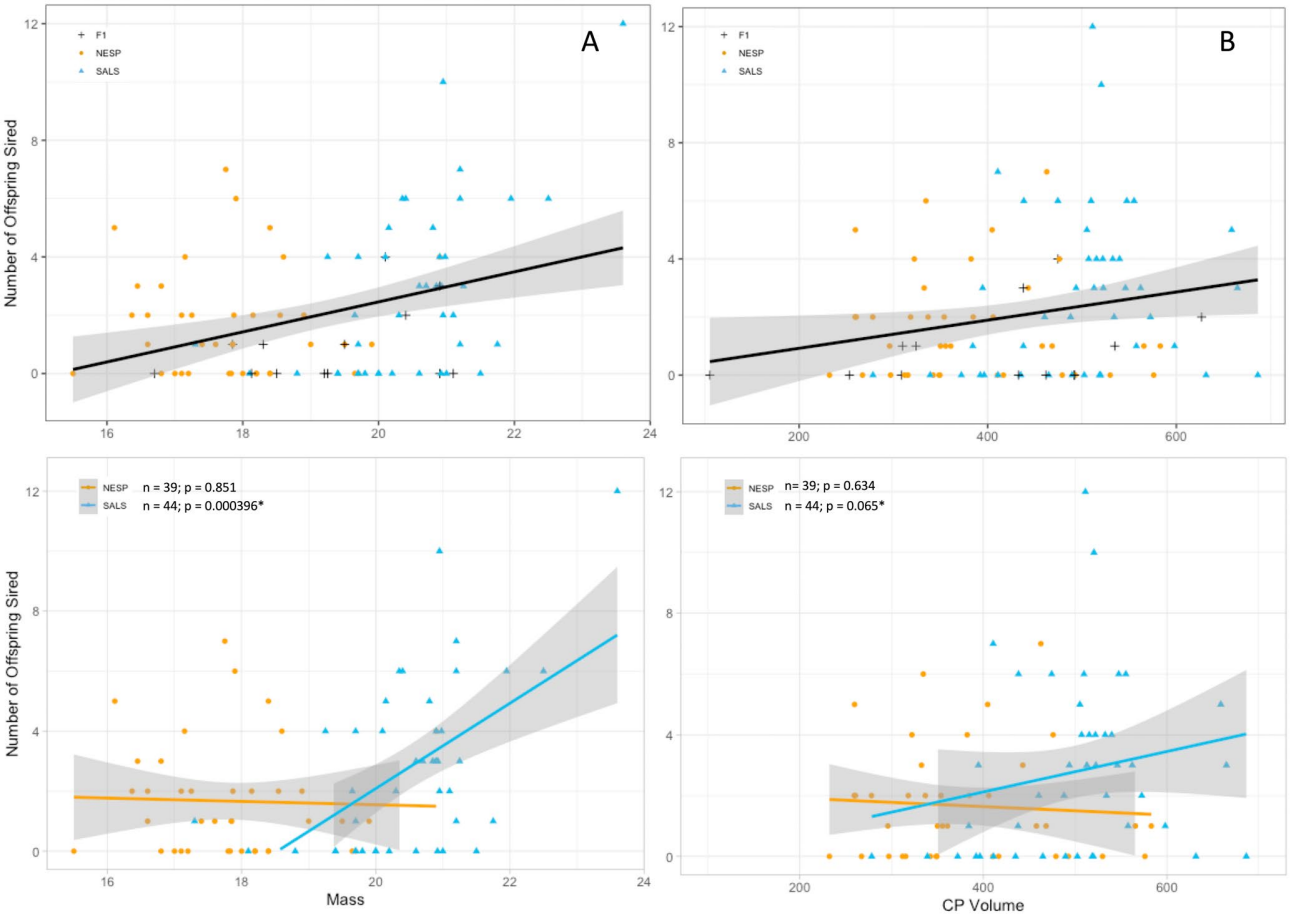
Relationship between the number of offspring sired and two informative male sexual traits: CP volume (B, D) and mass (A, C). Top two panels (A, B) show the trendline across all male genotypes with shaded 95% confidence intervals and species denoted with symbols/colors (black cross for hybrids, yellow circles for Nelson's sparrows, and blue triangles for saltmarsh sparrows). The bottom two panels (C, D) show trendlines within the two species separately (yellow dots are Nelson's sparrows and blue triangles are saltmarsh sparrow; hybrids removed from this analysis).

We modeled number of offspring sired in relation to four measured male sexual characteristics (CP volume, mass, fat scores, and muscle scores) to determine if male condition and competitive ability were positively correlated with reproductive success due to sexual selection (regardless of genotype). The top model (AIC_c_ = 369.1, *w*_i_ = 0.268) included only mass (*β* = 0.21; 95% CI = 0.08–0.34; Table 2). The next three models performed similarly (ΔAIC_c_ < 2) and included the addition of one of three covariates: muscle score (ΔAIC_c_ = 0.917), fat score (ΔAIC_c_ = 1.48), and CP volume (ΔAIC_c_ = 1.60); however, all 95% confidence intervals for beta coefficients of the additional covariates in those models overlap zero, suggesting they do not explain significantly more variance in offspring sired than mass alone (Table 2). Indeed, the top seven models all included mass with combinations of other covariates, but only mass was significant in each of these models (95% CI not overlapping zero; Table 2). The first model without mass (AIC_c_ = 373.3, ΔAIC_c_ = 4.18, *w*_i_ = 0.033) included only CP volume as a covariate, with a significant beta coefficient (β = 0.003, 95% CI = 0.0004–0.0005). The ΔAIC_c_ is high and the overall weight of the model low (AIC_c_ = 373.3, ΔAIC_c_ = 4.18, *w*_i_ = 0.033), suggesting relatively lower support for this model; however, this shows that CP volume may be predictive to a lesser extent in this system even if it explains much less variance in offspring produced than mass does. It is noteworthy that CP volume had a beta coefficient that did not overlap zero in all other models in which mass was absent and outperformed the global model when it was the only covariate in the model (Table 2). Thus, mass appears to be the best predictor of reproductive success in this system (with larger birds siring more offspring), with the possibility of CP volume predicting success to a lesser extent (males with larger CP volumes sire more offspring; Figure 2). Due to correlation found between CP volume and mass, this relationship could also be encapsulating some variation in overall size. Overall, there was no support for fat or muscle scores influencing the number of offspring sired across genotypes.

**TABLE 2:** Results of models assessing male condition and competitive ability as drivers of fitness (measured by the number of offspring sired by a male in one breeding season) across Nelson's, saltmarsh, and hybrid sparrows. Four metrics of male sexual characteristics—CP volume, mass, fat scores, and muscle scores—were modeled with a negative binomial regression. Models are listed sequentially by ΔAIC_c_ score, with associated beta coefficient values for each covariate (bold beta values denote informative predictors for which the 95% confidence interval did not overlap zero). Gray highlighted models include covariates we deemed as informative to reproductive success across all genotypes and were subsequently modeled within species.

Male fitness model	Fat score	CP volume	Mass	Muscle score	AIC_c_	ΔAIC_c_	Weight
{B0} + Mass	—	—	**0.208**	—	369.107	0.000	0.268
{B0} + Mass + Muscle	—	—	**0.222**	−0.213	370.025	0.917	0.170
{B0} + Fat + Mass	−0.198	—	**0.203**	—	370.596	1.489	0.128
{B0} + CP + Mass	—	0.001	**0.175**	—	370.704	1.596	0.121
{B0} + CP + Mass + Muscle	—	0.001	**0.191**	−0.205	371.756	2.649	0.071
{B0} + Fat + Mass + Muscle	−0.143	—	**0.217**	−0.186	371.905	2.797	0.066
{B0} + Fat + CP + Mass	−0.207	0.001	**0.168**	—	372.172	3.065	0.058
{B0} + CP	—	**0.003**	—	—	373.295	4.188	0.033
{B0} + Fat + CP + Mass + Muscle	−0.155	0.001	**0.184**	−0.176	373.624	4.517	0.028
{B0} + Fat + CP	−0.258	**0.003**	—	—	374.379	5.271	0.019
{B0} + CP + Muscle	—	**0.003**	—	−0.125	375.063	5.956	0.014
{B0}	—	—	—	—	376.269	7.162	0.007
{B0} + Fat + CP + Muscle	−0.235	**0.003**	—	−0.086	376.416	7.308	0.007
{B0} + Fat	−0.276	—	—	—	377.202	8.095	0.005
{B0} + Muscle	—	—	—	−0.111	378.098	8.991	0.003
{B0} + Fat + Muscle	−0.257	—	—	−0.061	379.293	10.186	0.002

Because we found mass and CP volume to be informative for predicting the number of offspring sired, we further modeled these two variables separately (in a univariate framework) within saltmarsh and Nelson's sparrows using negative binomial regression (for saltmarsh and Nelson's sparrows only, as the sample size of hybrids was too small to model independently), to determine if patterns held constant between species. For saltmarsh sparrow, we found results consistent with those across all genotypes. There was a significant positive relationship between mass and number of offspring sired (*β* = 0.52, *z* = 3.54, *p* = 0.0004; Figure 2), and a positive relationship between offspring sired and CP volume at the *p* < 0.10 alpha level (*β* = 0.52, *z* = 3.54, *p* = 0.065; Figure 2). For Nelson's sparrows, results differed from those found across genotypes, and neither mass (*β* = −0.031, *z* = −0.188, *p* = 0.851) nor CP volume (*β* = −0.001, *z* = −0.476, *p* = 0.634) were predictors of reproductive success (Figure 2). We also tested for correlations between CP volume and mass separately within species to ensure these patterns were not due to species differences alone, which showed that while CP volume and mass were correlated within saltmarsh sparrows (r = 0.36; *p* = 0.02), there was no correlation between CP volume and mass within Nelson's sparrows (r = 0.04; *p* = 0.80).

**FIGURE 3:**
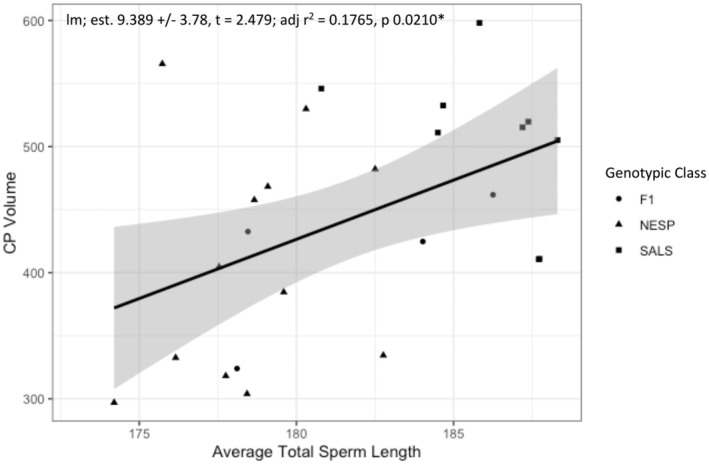
The linear regression of CP volume and total sperm length for a subset of 27 male sparrows from the 2016 and 2017 breeding seasons. Gray shading represents the 95% confidence interval with asterisk denoting significance at the 0.05 alpha level. Genotypic class is denoted with symbols (circles are hybrids, triangles are Nelson's sparrows, squares are saltmarsh sparrows).

**FIGURE 4:**
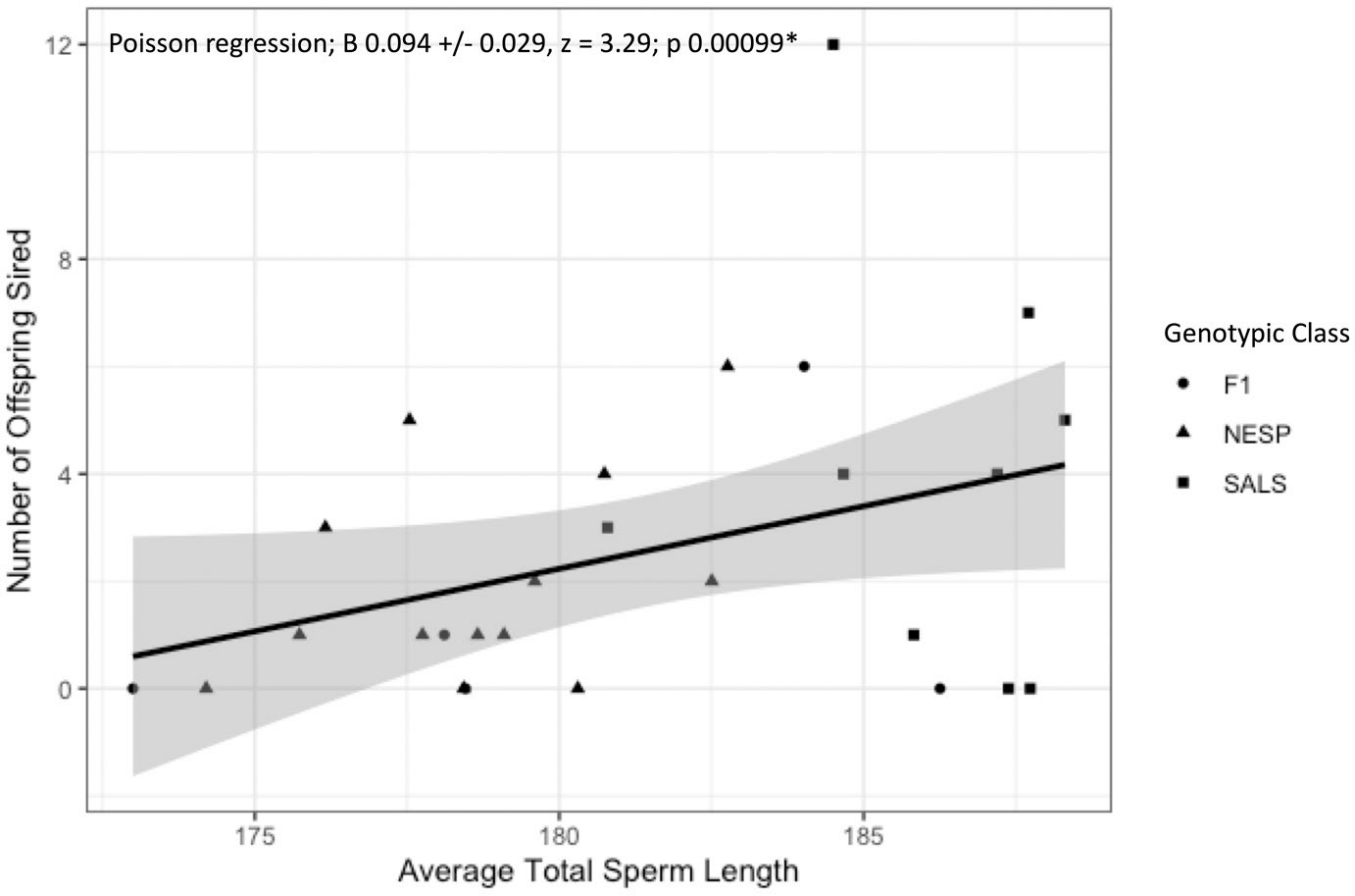
The poisson regression of number of offspring sired and total sperm length for a subset of 27 male sparrows from the 2016 and 2017 breeding seasons. Gray shading represents the 95% confidence interval with asterisk denoting significance. Genotypic class is denoted with symbols (circles are hybrids, triangles are Nelson's sparrows, squares are saltmarsh sparrows).

For a subset of sampled males for which we had data (*n* = 27), we found a significant positive linear relationship between CP volume and average sperm length of male sparrows across both species and hybrids (*β* = 9.4 ± 3.8, *t* = 2.5, *p* = 0.02; Figure 3), suggesting CP volume may be an appropriate proxy for sperm competition in this study. Although we did not test this relationship within species, we see similar patterns for Nelson's, saltmarsh, and hybrids (Figure 3). Additionally, patterns suggest Nelson's sparrows have smaller CP volume and average total sperm length than saltmarsh sparrows, although this was not tested formally due to small sample sizes (Figure 3). Finally, we also found a significant positive relationship between average total sperm length and the number of offspring a male sired across the species (*β* = 0.094 ± 0.029, *z* = 3.3, *p* = 0.00099; Figure 4).

### 3.3 Site‐ Specific Drivers of Fitness

To determine if drivers of male fitness differed between the sites within the center of the hybrid zone, we modeled the number of offspring sired in relation to four measured male condition metrics (mass, CP volume, fat, and muscle) separately at the coastal and inland site. We found that nothing predicted male reproductive success at the inland site, with the null model having the most support (AIC_c_ = 64.7, *w*_i_ = 0.216; Table A1). For the coastal site, the top model (AIC_c_ = 301.8, *w*_i_ = 0.283) included mass with a positive effect on number of offspring sired (*β* = 0.25, 95% CI = 0.16 to 0.03) and fat score with a negative effect (*β* = −0.34, 95% CI = −0.64 to −0.05; Table 3). The second most supported model (ΔAIC_c_ = 0.73) included reproductive success increasing with mass (*β* = 0.26, 95% CI = 0.18 to 0.35) and decreasing with muscle score (*β* = −0.26, 95% CI = −0.5104 to −0.0209; Table 3). There were two other models that had ΔAIC_c_ < 2, each with different combinations of muscle, fat, and CP volume; however, mass was the only consistently significant predictor across the top eight models (Table 3). Similar to the models for both sites combined (above), CP volume was a significant covariate in all the models without mass. There was very little support for those models with CP volume, however, with ΔAIC_c_ > 26, suggesting that mass is the most informative predictor, with fat and muscle also adding predictive power in some models and combinations (Table 3).

**TABLE 3:** Results of models assessing male condition and competitive ability (CP volume, mass, fat scores, and muscle scores) as drivers of fitness (number of offspring sired) at the coastal site. Models are listed sequentially by ΔAIC_c_ score, with associated beta coefficient values for each covariate (bold beta values denote informative predictors for which the 95% confidence interval did not overlap zero).

Coastal fitness model	Fat score	CP volume	Mass	Muscle score	AIC_c_	ΔAIC_c_	Weight
{B0} + Fat + Mass	**−0.3379**	—	**0.2482**	—	301.779	0.000	0.283
{B0} + Mass + Muscle	—	—	**0.2618**	**−0.2643**	302.507	0.728	0.196
{B0} + Fat + Mass + Muscle	−0.2455	—	**0.2521**	−0.1659	302.642	0.863	0.184
{B0} + Fat + CP + Mass	**−0.3402**	−0.0005	**0.2624**	—	303.729	1.951	0.107
{B0} + CP + Mass + Muscle	—	−0.0005	**0.2775**	**−0.2681**	304.406	2.627	0.076
{B0} + Fat + CP + Mass + Muscle	−0.2474	−0.0006	**0.2688**	−0.1714	304.564	2.785	0.070
{B0} + Mass	—		**0.2574**	—	304.850	3.071	0.061
{B0} + CP + Mass	—	−0.0004	**0.2702**	—	306.776	4.997	0.023
{B0} + Fat + CP	**−0.3786**	**0.0019**	—	—	328.098	26.320	0.000
{B0} + Fat + CP + Muscle	**−0.3304**	**0.0019**	—	−0.0921	329.889	28.110	0.000
{B0} + CP + Muscle	—	**0.0019**	—	−0.2069	331.540	29.761	0.000
{B0} + CP	—	**0.0018**	—	—	332.254	30.475	0.000
{B0} + Fat	**−0.3840**	—	—	—	332.840	31.061	0.000
{B0} + Fat + Muscle	**−0.3495**	—	—	−0.0773	334.673	32.894	0.000
{B0} + Muscle	—	—	—	−0.1762	337.018	35.239	0.000
{B0}	—	—	—	—	337.028	35.250	0.000

To ensure the lack of informative covariates found at the inland site was not a statistical artifact due to relatively small sample size of males at the inland site (*n* = 28) compared to that of the coastal site (*n* = 68), we subsetted the coastal dataset to 28 males (randomly selected) and re‐did the above modeling. Results were largely consistent with those from the full dataset, with both mass and muscle as informative predictors of reproductive success in the top two models (ΔAIC_c_ < 2), while fat was not a significant covariate in any of the models (Table A2). The most supported model (AIC_c_ = 110.5, *w*_i_ = 0.317) included mass with a positive effect (*β* = 0.195, 95% CI = 0.01 to 0.39) and muscle score with a negative effect on number offspring sired (*β* = −0.684, 95% CI = −1.23 to −0.19), while the second model (AIC_c_ = 111.9, ΔAIC_c_ = 1.42, *w*_i_ = 0.155) included only muscle with a negative effect (*β* = −0.53, 95% CI = −1.04 to −0.04) on reproductive success (Table A2). These results validate that the lack of informative predictors of reproductive success at the inland site is not a consequence of lack of statistical power due to sample size, but rather a true signal of observed patterns.

**FIGURE 5:**
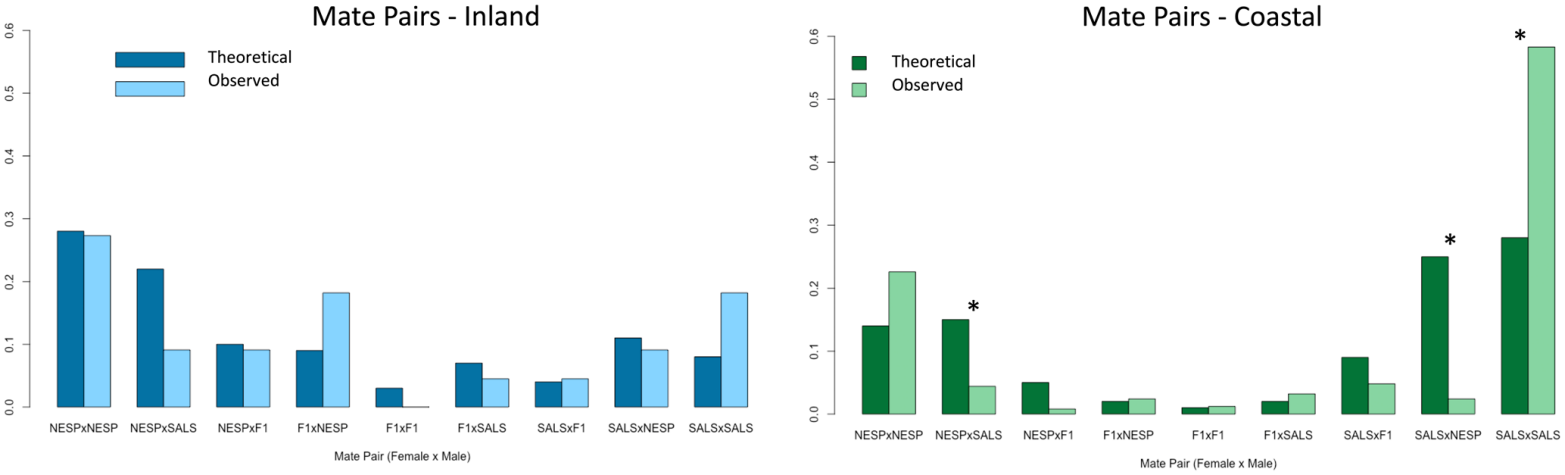
Observed (reconstructed from parentage analysis) and predicted (expectation from random mating based on observed distribution of male and female genotypes) distributions of mate pairings between Nelson's, saltmarsh, and hybrid sparrows across the 2016 and 2017 breeding seasons. Left panel includes birds from the inland site (light blue observed and dark blue predicted), while right panel includes birds from the coastal site (light green observed and dark green predicted).

**FIGURE A1:**
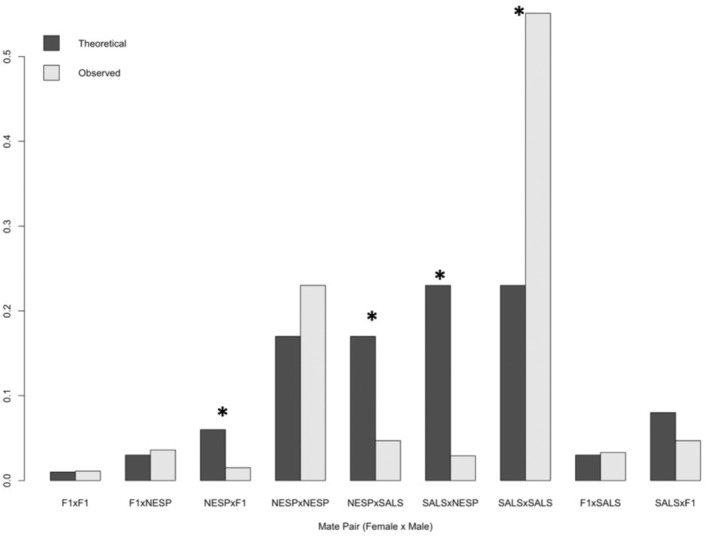
Observed (reconstructed from parentage analysis; light gray) and predicted (from random mating based on observed distribution of male and female genotypes; dark gray) number of mate pairings between Nelson's, saltmarsh, and hybrid sparrows across both sites during the 2016 & 2017 breeding seasons. Asterisk denotes significance between observed and predicted values at the 0.05 alpha level.

Finally, to determine if any single genotypic mate pairing dominated successful fertilization, we compared the proportion of successful mating attempts across nine possible mate pair categories to those predicted if random mating occurred from the existing genotypic distribution observed. This was performed both across sites and within sites separately to see if patterns were consistent across scales. Across sites, we observed a significant difference between the observed mate pairs and theoretical expectations (*p* = 0.0005), with fewer Nelson's sparrow female and hybrid male pairings than expected (*p* = 0.037), fewer Nelson's sparrow females and saltmarsh sparrow male pairings than expected (*p* = 0.00003), fewer saltmarsh sparrow females with Nelson's sparrow male pairings than expected (*p* = 7.2^−12^), and more saltmarsh sparrow females with saltmarsh sparrow males than expected (*p* = 5.6^−14^; Figure A1). These differences largely held true for the coastal site (*p* = 0.0005), with less Nelson's sparrow females and saltmarsh sparrow male pairings than predicted (*p* = 0.0003), fewer saltmarsh sparrow female and Nelson's sparrow male pairings than expected (*p* = 2.15^−13^), and more saltmarsh sparrow female and male pairings than predicted by random (*p* = 2.5^−11^; Figure 5). There was no significant difference between observed mate pairs and those predicted by random mating at the inland site (*p* = 0.945), indicating that mate pairs were random in regards to broad genotypic class (Figure 5). We do not see any clear patterns for one specific mate pair dominating all successful mating attempts across sites or within (Figure 5).

## 4 Discussion

### 4.1 Differential Fitness Across Genotypes

Saltmarsh sparrows males had greater reproductive skew than Nelson's sparrows and sired more offspring than both Nelson's sparrows and hybrids in sympatry, suggesting that interspecific reproductive competition may occur. Additionally, we found hybrid males sired similar numbers of nestlings as Nelson's sparrows, in contrast to prior work in the southern range of the hybrid zone, where hybrid males were found to sire lower numbers of offspring than either parental type (Walsh et al. 2018). Divergent mating behaviors could explain the observed difference in fitness and patterns of paternity among male saltmarsh, Nelson's, and hybrids. Nelson's sparrow males are known to mate guard and therefore likely have mating opportunities with fewer females than saltmarsh sparrow males, which use scramble competition search tactics (Greenlaw 1993; Shriver et al. 2007, 2010; Greenlaw and Post 2012). In line with these different strategies, we found saltmarsh sparrows sired offspring from a significantly higher number of nests than Nelson's sparrows and had comparably higher levels of multiple paternity across all nests.

Numerous males of each genotypic class were found not to sire any offspring across the 2‐year study period; however, variation in the number of offspring sired among males differed significantly in magnitude between the species, with greater reproductive skew in saltmarsh sparrows than Nelson's. This suggests there were a few saltmarsh sparrow males that were much more successful than the majority. This reproductive skew, where a small number of males produce high numbers of offspring, is indicative of scramble competition male mating tactics, where there are clear winners and losers in access to mates and/or fertilization rates (Andersson 1994). When the two species co‐occur in sympatry, the scramble competition mating behavior of saltmarsh sparrow males makes them better competitors overall. The larger size, as well as more active patrolling for mates may allow saltmarsh sparrows to outcompete smaller Nelson's sparrows, which may spend less time actively searching for mates and instead employ a mate‐guarding strategy (Greenlaw and Post 2012; Shriver et al. 2007). The mating strategy of Nelson's sparrows appears to be at a disadvantage in sympatric populations; however, it may be more effective in populations where there is no competition (allopatric) or sites with fewer saltmarsh sparrows and therefore reduced competition. Indeed, we saw less pronounced reproductive skew for both saltmarsh and Nelson's sparrow males at the inland study site, which is smaller and had more equal numbers of individuals of the two species. The coastal study site, on the other hand, had higher numbers of saltmarsh sparrows (53% adults of saltmarsh sparrow descent on coastal site compared to 31% inland) and higher skew in siring success. There were coastal saltmarsh sparrow males with very high success (> 7 offspring sired), but no Nelson's males with that rate of paternity on either of our sites. While Nelson's sparrows may be outcompeted in a saltmarsh sparrow dominated landscape, where access to females is heavily reliant on scramble tactics, the mate‐guarding strategy provides some success to Nelson's sparrows, albeit with fewer total offspring produced and with lower rates of multiple paternity than saltmarsh sparrows. Our results suggest that patterns of Nelson's sparrow paternity may be dependent on the density of saltmarsh sparrows within a site; however, it would be interesting to compare these patterns to those found in allopatric Nelson's populations. If patterns are partially mediated by relative species densities, we would expect even less reproductive skew and lower multiple paternity in the absence of saltmarsh sparrow competition (allopatry).

Levels of multiple paternity were high among all genotypic classes, but higher levels in saltmarsh than Nelson's sparrows are consistent with differences in the species' mating behavior. Remarkably high among avian species, the levels of multiple paternity observed in this study were consistent with, or higher than, those previously reported in the southern margins of the hybrid zone or in allopatric saltmarsh sparrow populations (Walsh et al. 2018; Hill et al. 2010). We found 35% of nests to have a different father for each chick and 73% of nests to have two or more fathers, compared to 33% and 60%, respectively, in allopatric saltmarsh sparrow populations (Hill et al. 2010). Of the nests we observed with a different father for each chick, the majority were saltmarsh sparrows (54%), followed by Nelson's sparrows (29%) and hybrids (18%). The pattern for saltmarsh sparrows is similar to that found in the southern range of the hybrid zone, with 57% of saltmarsh sparrow nests reported to have a different father for each chick; however, it is lower for Nelson's sparrows, with 75% reported to have a different father for each chick (Walsh et al. 2018). Differences in findings between these two studies may be due to sample size, as the study of Walsh et al. (2018) had very small samples sizes of pure Nelson's sparrows (only eight broods total). Our results from the center of the hybrid zone with more equal species abundances provide a more robust view of paternity patterns in sympatric Nelson's sparrow populations. Here we provide further evidence for high levels of multiple paternity despite mate‐guarding tendencies, which had been hypothesized to limit extreme multiple paternity. Alternately, levels of multiple paternity indeed may be higher in the southern end of the range where Nelson's sparrows are rare, if inter‐specific matings by saltmarsh sparrow males contribute to and inflate the levels of multiple paternity of Nelson's sparrow broods. Accordingly, the relatively lower levels of multiple paternity we observed for Nelson's sparrows in the center of the hybrid zone may reflect relatively higher success of the mate‐guarding strategy in the presence of fewer Saltmarsh Sparrows. Understanding the levels of multiple paternity and reproductive skew of Nelson's sparrow in allopatry would be important to determine in future work, to evaluate these hypotheses.

### 4.2 Pre‐ and Post‐Copulatory Drivers of Male Fitness Vary Across a Small Spatial Scale

Across saltmarsh, Nelson's, and hybrid males, we found that the best predictor of male reproductive success was body weight, suggesting pre‐copulatory sexual selection may contribute to patterns of gene flow and hybridization between the species. Competition is an important determinant of mating success, especially for individuals with polygynous mating systems where reproductive success is skewed towards dominant individuals (Clutton‐Brock 2007; Moller 1988). Male–male competition between saltmarsh and Nelson's sparrows could come in the form of aggressive behavior among males that may allow for the dominant bird to copulate with more females (Darwin 1871; Andersson 1994). It may also come in the form of time spent searching for mates, which may allow for a male to copulate with a female before others and gain a competitive edge (Hasselquist and Bensch 1991; Schwagmeyer and Woontner 1986). Male–male competition will often select for large body size (Greenlaw 1993; Andersson 1994), which is known to be important for avian competition (Andersson 1994), correlating with reproductive success in numerous birds species (Chastel et al. 1995; Dyrcz et al. 2005; Sanchez‐donoso et al. 2018). The larger saltmarsh sparrow males may out‐compete smaller Nelson's male or hybrids where access to mates is limited. It is unlikely that one sex controls mate choice entirely—interactions between male and female choice may ultimately determine mate success. Females may solicit competition among males and make choices based on displayed dominance (Andersson 1994). Indeed, saltmarsh sparrow females have been known to solicit mating during nest building as well as to prevent forced mountings by males by fighting or aggressive calls (Greenlaw and Post 2012), suggesting some female control exists in this system. Females may also be actively choosing to accept matings with saltmarsh sparrow males that are larger in body size. These dynamics are not consistent throughout the hybrid zone, however, as mass was not found to be predictive of reproductive success in the southern range of the hybrid zone (Walsh et al. 2018). This suggests that patterns of paternity and drivers of reproductive success vary spatially throughout the hybrid zone.

Although pre‐copulatory traits, such as mass, appear to better predict interspecific reproductive success in this system, we also found evidence supporting post‐copulatory sexual selection and sperm competition between the species. Larger CP volumes and sperm length in saltmarsh sparrows than Nelson's sparrows, as well as evidence for increased reproductive success with CP volume in the absence of mass (and sperm length for the subset of males which had morphology data), suggests that sperm competition is likely a factor contributing to fertilization rates and male reproductive success in this system. Body size is likely the best predictor of reproductive success since it represents access to potentially fertile females through pre‐copulatory competition, while CP volume and sperm competition is only secondarily important, since it can only act post‐copulation.

Post‐copulation fertilization success can be influenced by either male–male competition or female choice in the form of sperm competition and cryptic female choice (Parker 1970; Birkhead 1998). Sperm competition is higher in males of species with polygynous mating systems, as measured by traits such as testes length, CP volume, or ejaculate quality and quantity (Briskie 1993; Moller 1988). Further, CP volume has been found as a proxy for sperm production, sperm velocity, and resulting fertilization success in birds (Laskemoen et al. 2008, 2010; Peer et al. 2000; Tuttle et al. 1996). If saltmarsh sparrows have higher sperm production or velocity than that of Nelson's sparrows, eggs may be preferentially fertilized by saltmarsh sparrow males outcompeting sperm of Nelson's. Females may also have control over post‐copulatory mate choice. Since we did not account for any cryptic female choice in this study, we therefore cannot eliminate the effect of female sparrows biasing fertilization rates between males of differing genetic makeup.

Traits that determined interspecific success were consistent with those found to predict mating success in saltmarsh sparrow (mass and CP volume to a lesser extent); however, none of our measured male condition or competitive ability traits predicted success in Nelson's sparrow. This may be explained by the Nelson's sparrow's divergent mating strategy, whereby size may not matter as much due to active mate guarding of females. Smaller body size may also provide advantages in acrobatic performance in their characteristic flight displays to attract females (Walsh, Shriver, et al. 2015; Walsh et al. 2018). Mating behavior such as length of singing period or active display period may be more important to Nelson's sparrows than overall size in attracting females (Greenlaw 1993; Shriver et al. 2007, 2010). Additionally, the frequency and length of mate guarding may be important in predicting success. This may act to ensure a male's sperm does not get replaced by another male and increase the odds of fertilization. There also may be other secondary sexual characteristics driving sexual selection that were not accounted for in this study. In particular, known differences in male song and frequency of mating display/singing should also be considered (Greenlaw 1993; Shriver et al. 2005, 2007, 2010).

Drivers of fitness not only differed between species, but also across sites. While none of the measured pre‐ or post‐copulatory traits predicted male fitness at the small inland site, patterns of male reproductive success at the coastal site were similar to those across all individuals within the center of the hybrid zone (even when sample size differences among sites were considered), signaling sexual selection is operating differently across sites. The coastal site is larger in size with a higher density of birds and different makeup of genotypic classes, with more saltmarsh and backcrossed saltmarsh individuals than pure or backcrossed Nelson's as compared to the inland site (Maxwell et al. 2021). Reproductive success at the coastal site was predicted best by traits that involve dominance or competitive edge in a scramble mating interaction, such as increasing size of the male, with some evidence for increased fitness with a decrease in fat and muscle scores. This suggests that most successful males at the coastal site are bigger and likely expending more energy finding, competing for, and copulating with females (Vehrencamp et al. 1989), leading to reduced fat stores or observed muscle for a given mass. As observed across all males, there was some limited evidence for CP volume and sperm competition influencing male fitness; however, this was only secondary to male size, further exemplifying precopulatory processes dominating which males can mate and extend their postcopulatory competitive advantage. None of these traits predicted male success at the smaller inland site, which also appeared to have more equal relative reproductive success (less skew), suggesting differing selective pressures. Saltmarsh sparrows may be dominating the mating system at large, high‐density sites where interspecific competition is high and access to mates may be limited. Nelson's sparrows appear to be more successful when interspecific competition is lower, which may not necessarily indicate an inferior competitor, but may reflect the relative success of the mate guarding tactic, which may only confer fitness benefits on sites with relatively lower densities of saltmarsh sparrows.

### 4.3 Implications for Hybrid Zone Dynamics

Variance in fitness levels across species and sites have implications for extent and directionality of hybridization. Competitive interactions may lead to geographic or genetic displacement of the inferior competitor—Nelson's sparrows—causing the hybrid zone to appear more saltmarsh‐sparrow‐like over time, as illustrated in the hermit ( *Setophaga occidentalis* ) and Townsend's ( *S. townsendi* ) warbler hybrid zone (Pearson 2000). Hybridization may also benefit male Nelson's sparrows in this system by increasing their fitness or competitive ability through the acquisition of saltmarsh sparrow alleles. While this could represent a competitive advantage and subsequently increase rates of hybridization or drive patterns of introgression, successful interspecific offspring production is relatively rare, suggesting reinforcement through assortative mating may limit hybridization in this system. For example, it is hypothesized that sperm length may act as a postcopulatory prezygotic reproductive barrier which has co‐evolved with the size of specialized female storage tubules between the species (Cramer et al. 2021). Indeed, we saw few hybrids, reduced survival of hybrid females in support of Haldane's Rule, and nearly equal backcrossing in both the saltmarsh and Nelson's direction in the center of the hybrid zone (Maxwell et al. 2021), suggesting that mechanisms exist to maintain species boundaries and minimize asymmetric introgression towards one parental species or the other.

Drivers of reproductive success and resulting gene flow between saltmarsh and Nelson's sparrows vary on a fine spatial scale along a patchy mosaic of microhabitat in the center of the hybrid zone. While patterns of successful mate pairs across at the population‐level in the center of the hybrid zone are consistent with assortative mating (more within species mate pairs than predicted by random), this pattern is driven by dynamics at the coastal site, while the inland site had mate pair patterns that were not different from those predicted by random mating across the available adult genotypes. These patterns of differing selective pressures and their relative strength across a fine spatial scale in the center of the hybrid zone corroborate previous findings that highlighted the context‐dependent nature of the factors influencing gene‐flow dynamics and hybrid zone structure (Maxwell et al. 2021).

Site‐specific characteristics in the form of bird density or population size, distribution of genotypes and competitive landscape, and/or microhabitat may influence the level of sexual selection and resulting patterns of gene flow between saltmarsh and Nelson's sparrows in this mosaic hybrid zone. We found the larger, coastal site to have higher levels of reproductive skew (suggesting higher male competition) and assortative mating than the smaller, inland site which was dominated by random mating and lower rates of skew between species and individuals. The two sites reside within the center of the hybrid zone but differ along a coastal‐inland habitat gradient. While we did not test for the influence of exogenous selection on mate pair patterns and gene flow, is it possible that environmental heterogeneity along a continuum of brackish to coastal tidal marshes may generate a selective gradient and habitat‐specific mating opportunities, which could partially determine the nature of species boundaries, as seen in other studies (McKenzie et al. 2017; Shurtliff et al. 2014). Indeed, directional hybridization may also be adaptive in certain environments where inheritance of competitive traits from the dominant parent may provide a selective advantage and drive patterns of hybridization, as is seen in hybrid tadpoles ( *S. bombifrons* and *S. multiplicate*), where females are more likely to mate with one parental species in certain environmental conditions to which that parental species has a competitive advantage (Pfennig and Simovich 2002). Differences in spatial groupings of individuals and the differing density of genotypic classes between sites may also influence variation in rates of genetic exchange through access to potential mates or the level of interspecific competition. If the inland‐coastal patterns observed in this study are indicative of larger habitat dynamics across this mosaic hybrid zone, then the majority of hybrids are produced on inland sites, indicative of a breakdown of isolating mechanisms in small populations. This suggests that small inland sites could play a disproportionate role in maintaining hybridization and interspecific gene flow between the species, consistent with the patterns of introgression observed across 26 sites spanning the hybrid zone (Walsh, Shriver, et al. 2015).

We saw processes influencing genetic exchange differ on a very small scale between sites within the center of the hybrid zone, suggesting these dynamics are not always stable, but dependent on site‐specific characteristics, as seen in other studies (Shurtliff et al. 2014; Zonana et al. 2021). Factors including pre‐ and post‐copulatory male competitive traits, differential fitness, and reduced fitness of hybrid females (Maxwell et al. 2021) all appear to play a role in hybrid zone dynamics. This adds to a growing body of literature that shows hybrid zones with semipermeable barriers are often maintained by a complex interaction of multiple isolating mechanisms (Arias et al. 2012; McKenzie et al. 2017).

## Author Contributions

**Logan M. Maxwell:** conceptualization (equal), formal analysis (lead), investigation (lead), methodology (equal), project administration (equal), writing – original draft (lead), writing – review and editing (equal). **Jennifer Walsh:** conceptualization (equal), formal analysis (supporting), methodology (equal), writing – review and editing (equal). **Brian J. Olsen:** conceptualization (equal), methodology (equal), writing – review and editing (supporting). **Adrienne I. Kovach:** conceptualization (equal), funding acquisition (lead), methodology (equal), project administration (lead), supervision (lead), writing – review and editing (equal).

## Ethics Statement

This research was conducted in accordance with the Institutional Animal Care and Use Committee of the Universtiy of New Hampshire (160503) and all capture, banding, and blood sampling was conducted with State of Maine and Federal permits (#24045).

## Conflicts of Interest

The authors declare no conflicts of interest.

## Supporting information


Data S1.

Data S2.

## Data Availability

Analyses reported in this study can be replicated using male reproductive success data and measured sexual traits (Supporting Information Data S1), as well as mate pair data from genetic parentage reconstruction (Supporting Information Data S2) which are included as excel sheets in Supporting Information. Sperm measurements used in this study are published and available with Cramer et al. (2021).

## Appendix A

**TABLE A1:** Results of negative binomial regression models assessing male condition and competitive ability (CP volume, mass, fat scores, and muscle scores) as drivers of fitness (number of offspring sired) at the inland site. Models are listed sequentially by ΔAIC_c_ score.

Inland fitness model	Fat score	CP volume	Mass	Muscle score	AIC_c_	ΔAIC_c_	Weight
{B0}	—	—	—	—	64.7414	0.0000	0.2161
{B0} + Mass	—	—	−0.2505	—	64.8922	0.1508	0.2004
{B0} + Fat + Mass	−0.4062	—	‐0.2710	—	66.6066	1.8651	0.0851
{B0} + Fat	−0.2878	—	—	—	66.6536	1.9121	0.0831
{B0} + Muscle	—	—	—	−0.2012	66.8335	2.0921	0.0759
{B0} + CP	—	−0.0002	—	—	67.0549	2.3134	0.0680
{B0} + Mass + Muscle	—		−0.2447	−0.1167	67.3319	2.5905	0.0592
{B0} + CP + Mass	—	0.0003	−0.2552	—	67.3931	2.6517	0.0574
{B0} + Fat + Muscle	−0.3624	—	—	−0.2846	68.7244	3.9830	0.0295
{B0} + Fat + CP + Mass	−0.5612	0.0017	−0.3068	—	68.9620	4.2206	0.0262
{B0} + Fat + Mass + Muscle	−0.4555	—	−0.2631	−0.2089	69.0945	4.3531	0.0245
{B0} + Fat + CP	−0.3128	0.0003	—	—	69.1553	4.4139	0.0238
{B0} + CP + Muscle	—	−0.0003	—	−0.2071	69.3318	4.5903	0.0218
{B0} + CP + Mass + Muscle	—	0.0002	−0.2476	−0.1093	70.0640	5.3226	0.0151
{B0} + Fat + CP + Muscle	−0.3915	0.0003	—	−0.2838	71.4399	6.6985	0.0076
{B0} + Fat +CP + Mass + Muscle	−0.5827	0.0015	−0.2927	−0.1680	71.7787	7.0373	0.0064

**TABLE A2:** Models assessing male condition and competitive ability (CP volume, mass, fat scores, and muscle scores) as drivers of fitness (number of offspring sired) at the coastal site with a reduced dataset (28 random males) to test for statistical power. Models are listed sequentially by ΔAIC_c_ score, with associated beta coefficient values for each covariate (bold beta values denote informative predictors for which the 95% confidence interval did not overlap zero).

Sample size test (coastal)	Fat score	CP volume	Mass	Muscle score	AIC_c_	ΔAIC_c_	Weight
{B0} + Mass + Muscle	—	—	**0.1953**	−**0.6841**	110.4499	0.0000	0.3168
{B0} + Muscle	—	—	—	−**0.5277**	111.8741	1.4243	0.1554
{B0} + CP + Mass + Muscle	—	−0.0014	0.2662	−**0.7002**	112.9586	2.5088	0.0904
{B0} + Fat + Mass + Muscle	0.2076	—	**0.2090**	−**0.7921**	113.0301	2.5802	0.0872
{B0}	—	—	—	—	113.3781	2.9283	0.0733
{B0} + CP + Muscle	—	0.0016	—	−**0.5749**	113.4492	2.9993	0.0707
{B0} + Fat and Muscle	0.0823	—	—	−**0.5621**	114.5512	4.1013	0.0408
{B0} + Mass	—	—	0.1150	—	114.6282	4.1783	0.0392
{B0} + Fat	−0.2251	—	—	—	115.3877	4.9379	0.0268
{B0} + CP	—	0.0010	—	—	115.4674	5.0175	0.0258
{B0} + Fat + CP + Mass + Muscle	0.1606	−0.0012	0.2652	−**0.7795**	115.9884	5.5385	0.0199
{B0} + Fat + CP + Muscle	0.2082	0.0019	—	−**0.6759**	116.0851	5.6352	0.0189
{B0} + Fat + Mass	−0.2308	—	0.1171	—	116.7863	6.3364	0.0133
{B0} + CP + Mass	—	−0.0006	0.1460	—	117.2904	6.8406	0.0104
{B0} + Fat + CP	−0.1957	0.0009	—	—	117.8264	7.3765	0.0079
{B0} + Fat + CP + Mass	−0.2701	−0.0011	0.1735	—	119.5256	9.0758	0.0034
